# Diabetes‐related complications: Which research topics matter to diverse patients and caregivers?

**DOI:** 10.1111/hex.12649

**Published:** 2017-11-22

**Authors:** Maman Joyce Dogba, Mylène Tantchou Dipankui, Selma Chipenda Dansokho, France Légaré, Holly O. Witteman

**Affiliations:** ^1^ Department of Family and Emergency Medicine Faculty of Medicine Laval University Quebec City QC Canada; ^2^ Office of Education and Professional Development Faculty of Medicine Laval University Quebec City QC Canada; ^3^ Centre Hospitalier Universitaire de Québec (CHU de Québec) Research Centre [Health of populations and best health practices axis] Quebec City QC Canada

**Keywords:** diabetes, diabetes‐related complications, participatory research, patient engagement, patient involvement, patient‐oriented research, stakeholder voice, under‐represented populations

## Abstract

**Background:**

Diabetes is a chronic disease with increasing prevalence worldwide. Although research has improved its treatment and management, little is known about which research topics matter to people living with diabetes, particularly among under‐represented groups.

**Objectives:**

To explore the importance of research topics among a diverse range of people living with any type of diabetes or caring for someone living with any type of diabetes.

**Methods:**

We used a convergent mixed‐method design with quantitative and qualitative aspects. We surveyed a national sample of people living with diabetes and caregivers of people with diabetes, asking them to rate the importance of 10 predetermined important research topics. We also held three focus groups in two major cities to explore research concerns of people who are under‐represented in research.

**Results:**

469 adults (57% men, 42% women) in Canada completed the online survey, indicating that all 10 areas of research mattered to them, with the highest ratings accorded to preventing and treating kidney, eye and nerve complications. Fourteen individuals participated in three focus groups and similarly noted the importance of research on those three complications. Additionally, focus group participants also noted the importance of research around daily management. No new topics were identified.

**Conclusions:**

This study confirmed the importance of research topics among a population of people living with or caring for someone with diabetes. Findings from this study were used to inform the vision for Diabetes Action Canada—a pan‐Canadian Strategy for Patient‐Oriented Research (SPOR) Network on diabetes and its complications.

## 1 INTRODUCTION

Diabetes is a chronic disease with increasing prevalence worldwide.^1^ In 2014, an estimated 422 million adults, representing 8.5% of the global population, were living with diabetes.^2^ The economic burden of this disease and its complications account for a growing proportion of local and national budgets.^3^, ^4^ For individuals, diabetes has negative psychosocial consequences that diminish quality of life.^5^ While research has improved the treatment and management of diabetes and increased longevity,^6^ mismatches between the focus of research and what matters to patients may lead to research waste.^7^, ^8^ Involving patients in the early stages of research is the first step in reducing such waste, as it helps increase the relevance of topics studied regarding such chronic diseases as diabetes.^9^ People living with chronic diseases may develop a high degree of expertise which can provide new insight into how to improve their conditions and self‐care.^10^, ^11^ The patient perspective may complement that of the clinician and researcher by providing a more holistic interpretation of health and the experience of a health condition.^11^

When seeking to involve patients as partners in research, it is critical to avoid reproducing or even exacerbating health inequities. Major disparities persist in the diagnosis, treatment, disease management and health outcomes of groups such as ethnic minorities, immigrants, people living in poverty, people whose mental health require regular follow‐up with a psychiatrist and seniors, all of whom are more vulnerable to diabetes‐related complications.^12^, ^13^, ^14^, ^15^, ^16^ In spite of these continuing disparities, minority groups continue to be under‐represented in research, and engagement in defining research questions is no exception. Additionally, there is little guidance on how to facilitate the full participation of members of these groups in setting research priorities.^17^, ^18^ For example, although previous research in the United Kingdom has identified research priorities among people living with type 1 diabetes, those involved were predominantly white and female.^19^

This study aimed to involve a national sample of people living with diabetes and caregivers of people living with diabetes in rating the importance of research topics around diabetes‐related complications. We further sought to capture the perspectives of people who are under‐represented in research. Our primary research question was as follows: What topics are most important to people living with or caring for someone living with diabetes regarding disease‐related complications as a means to help better orient future research priorities?

## 2 METHODS

### 2.1 Study design

We used a multipronged mixed‐methods (QUAN + QUAL) approach with a convergent design^20^ to capture what was important to people living with diabetes and caregivers regarding research on diabetes‐related complications.^21^, ^22^ According to the convergent mixed‐methods design, quantitative and qualitative methods are complementary during data collection, data analysis or both. In our case, we combined^21^ the quantitative and qualitative data after we completed both sets of data collection. The study consisted of two components accordingly: (i) quantitative: an anonymous online survey to poll a national sample of people living with diabetes or caregivers of people living with diabetes on the importance of 10 predetermined research topics; and (ii) qualitative: holding focus groups with people living with diabetes who are members of under‐represented groups, in order to explore the views and experiences of those predicted to be under‐represented in the online survey.^23^

### 2.2 Research ethics

This study was approved by the Research Ethics Board of the Centre Hospitalier Universitaire de Québec (Quebec City, approval #: 2016‐2578). In agreeing to follow the link and take the online survey, participants provided implied consent. No survey questions were mandatory, meaning that respondents could skip questions if they wished. No attention filter was included. Prior to each focus group, we described the project and allowed participants to ask questions. Verbal consent of focus group participants was recorded.

### 2.3 Procedures

#### 2.3.1 Online survey

The online survey included questions on socio‐demographics, the person's experience with diabetes‐related complications and analog scales to rate the importance of 10 pertinent disease‐related complication research topics. These topics were identified in the literature describing previous priority‐setting exercises conducted with people living with type 1 diabetes,^19^ and via email consultation with researchers, clinicians, representatives of patient organizations, caregivers and patient partners as part of a 6‐month funding application planning process.

Demographic data gathered from participants included: age, gender, ethnicity, income and education levels, geographical location and country of birth (inside or outside of Canada). Prior to finalizing the survey, all survey questions were iteratively reviewed by a person living with type 1 diabetes, a person living with type 2 diabetes, and a parent of a child with type 1 diabetes. The survey also contained three validated scales^24^, ^25^, ^26^, ^27^, ^28^ to measure fear or distress associated with living with diabetes and its complications (see Appendix S1). These scales were included because we believed that fear or distress might influence how individuals rate the importance of research topics relative to the levels of fear they experience regarding these complications. If we were to observe large variations in ratings of importance, these data would allow us to explore potential reasons for the variation. The survey also included comment boxes where participants could provide additional information, including an open‐ended question asking for their ideas on additional topics concerning diabetes and diabetes‐related complications that require more research.

#### 2.3.2 Survey participants

Over a 3‐day period in September 2015, we recruited participants through Qualtrics online sampling services.^29^ To be included in the study, participants had to be living in Canada, aged ≥18 years, living with type 1 or type 2 diabetes, or caring for a child or an adult with diabetes and able to complete the survey in English or French. To ensure demographic diversity and offset variations in response rates, we established desired quotas based on gender (50/50 men and women), type of diabetes and relationship with diabetes (people with diabetes themselves, parents of children with diabetes, caregivers for adults with diabetes). We could not put quotas in place regarding ethnicity due to sampling constraints. In keeping with standard amounts for surveys administered by panel services, participants who completed the survey received $1.00‐$1.50 in compensation for their time answering our questions. We aimed for approximately 500 respondents. This target was selected as an achievable sample size that would allow for a broad sample of respondents and aligned with previous, similar research that sought feedback from 583 people living with diabetes about research questions they would like to see addressed.^19^

#### 2.3.3 Focus groups

Members of some groups may be less likely to complete online surveys, and thus, be under‐represented in survey‐based research. Therefore, we held 3 focus groups with patients and caregivers who were members of such groups.^30^ To ensure variation in perspectives, we partnered with community organizations working with seniors, economically disadvantaged people, immigrants and people whose mental health requires follow‐up with a psychiatrist.^31^, ^32^, ^33^ Two experienced qualitative researchers (MJD and MDT) conducted the focus groups using an established protocol. During the focus groups, patients were invited to discuss their experience with diabetes and its related complications, their perspectives and their concerns about the long‐term complications of diabetes. Participants also explained why, in their view, the concerns raised should be investigated by researchers.

#### 2.3.4 Focus group participants

We used a convenience sample of members of under‐represented populations in the province of Quebec. We recruited focus group participants through three community‐based organizations that provide services to seniors, immigrants and people whose mental health requires regular follow‐up with a psychiatrist. To be eligible to participate in the focus groups, participants needed to be: living in Canada, aged ≥18 years, living with type 1 diabetes, type 2 diabetes or caring for a person with diabetes, and able to understand and express themselves in French. Participants who were unable to comfortably express themselves in French were excluded from the study.

To recruit participants, the organizations circulated information about the study to its clients or members. Interested participants contacted the research associate either by email or by phone. The research associate contacted all potential participants to explain the study, assess their eligibility, answer questions and discuss logistics. A reminder call and/or email was sent to all participants 2 days prior to the scheduled focus group to confirm the time and location.

We held the three focus groups at times convenient for participants. Furthermore, to increase accessibility, the focus groups were held in the offices of the partnering community organizations; a common practice when working with members of vulnerable populations.^34^ We conducted two focus groups in Quebec City: (i) seniors; and (ii) people whose mental health requires regular follow‐up with a psychiatrist. We conducted the third in Montreal with a group of immigrants. Each focus group was audio‐recorded and lasted between 70 and 90 minutes. Participants received $50 in appreciation for their time and 10$ for transportation.^19^

### 2.4 Data analysis

Our interest in conducting both qualitative and quantitative portions was to ensure inclusion of diverse perspectives. In other words, while research often uses quantitative and qualitative methods to collect different types of data from the same population to inform a research question, we used different methods to collect data from groups both more and less likely to participate in different types of research, in an attempt to capture more representative results. Therefore, we carried out quantitative and qualitative analyses separately before bringing both parts together. Our first step was to conduct descriptive statistics using SPSS version 22 (Armonk, NY, USA: IBM Corp.) to measure central tendency and examine the range of variation in responses to our questions about the importance of 10 important diabetes research areas. We recorded focus group discussions and transcribed them verbatim. We performed a six‐stage thematic analysis^35^, ^36^ using NVivo qualitative analysis software (QSR International Pty Ltd. Version 10, 2012). We started by generating initial codes and themes, and inductively refining these themes based on the data. MTD analysed focus group data under the guidance of MJD. The codes were labelled with short phrases using the words of participants. Then, MTD sorted codes into potential themes and collated all relevant coded data extracts within the identified themes and subthemes. During this analysis, the codes, themes and subthemes were revised and refined. We used field notes^37^ to validate and complete the information gathered during the focus groups. After separate analyses were completed, we combined the findings from each study to analyse how complementary or contradictory they were. We additionally examined how focus group findings could improve our interpretation of the statistical analysis.

## 3 RESULTS

### 3.1 Characteristics of participants

#### 3.1.1 Online survey

Of the 500 participants surveyed, 31 were excluded from our analyses because they either completed the survey in a time deemed too fast to provide thoughtful answers (ie, 10 minutes or less) or because their responses were inconsistent with the questions. The remaining 469 participants were 57% men, had a mean age of 44 (SD = 15), came from across the 10 provinces and 3 territories of Canada, and represented a broad range of educational backgrounds and income levels. In line with our concerns and predictions about representation, participants predominantly identified as White or Caucasian (93%). Participants completed the survey in English (78%) or French (22%) and were either living with diabetes (96%) and/or caring for a child (<1%) or adult with diabetes (3%). Ten percent (10%) of participants were dealing with type 1 diabetes; 89% with type 2 diabetes; and 1% with another or unknown type. Median time living with diabetes was 19.5 years for type 1 diabetes (IQR 9.8‐30.0 years) and 8.0 years for type 2 diabetes (IQR 4.0‐15.0 years.) (See Table 1 A,B).

**Table 1:** Online Survey Data

(A) Socio‐deomgraphic characteristics of participants (N=469)	
Age (y): Mean (SD)	43.6 (14.6)
Sex: n (%)	
Male	268 (57.1)
Female	197 (42.0)
Other	1 (0.3)
Skipped answer	3 (0.6)
Race^a^: n (%)	
Aboriginal	6 (1.3)
Asian	18 (3.8)
Black	3 (0.6)
Hispanic	2 (0.4)
Middle Eastern	2 (0.4)
White or Caucasian	433 (92.5)
Other	8 (1.7)
Prefer not to say	1 (0.2)
Currently living in: n (%)	
British Columbia	49 (10.5)
Alberta	29 (6.2)
Saskatchewan	14 (3.0)
Manitoba	25 (5.4)
Ontario	173 (37.5)
Quebec	126 (27.1)
New Brunswick	18 (3.9)
Nova Scotia	23 (4.9)
Prince Edward Island	3 (0.6)
Newfoundland & Labrador	4 (0.9)
Yukon	1 (0.2)
Northwest Territories	0
Nunavut	0
Other	0
Prefer not to say	0
**Missing**	**4**
Born: n (%)	
In Canada	403(85.9)
Outside Canada	53 (11.3)
**Missing**	**13 (2.8)**
Education: n (%)	
None	0
Elementary school	8 (1.7)
High school	101 (21.5)
Trade school	46 (9.8)
Some post‐secondary	92 (19.6)
Associate's degree	100 (21.3)
Bachelor's degree	82 (17.5)
Graduate or professional degree	27 (5.8)
I prefer not to say	4 (0.9)
**Missing**	**9 (1.9)**
Income: n (%)	
Less than $20 000/y	64 (13.6)
$20 000‐39 000/y	110 (23.5)
$40 000‐59 000/y	94 (20.0)
$60 000‐79 000/y	65 (13.9)
$80 000‐99 000/y	51 (10.9)
$100 000 or more/y	44 (9.4)
I prefer not to say	38 (8.2)
**Missing**	**3 (0.6)**
Language of survey: n (%)	
English	357 (76.1)
French	103 (22.0)
Do not know	9 (1.9)

(B) Participants’ experience with diabetes	
Person affected by diabetes: n (%)	
Self, type 1	38 (9.2)
Child, type 1	1 (0.2)
Adult loved one, type 1	2 (0.5)
Self, type 2	354 (85.9)
Child, type 2	1 (0.2)
Adult loved one, type 2	10 (2.4)
**Other or don't know**	**6 (1.5)**
Years with diabetes: Mean (SD)	
Type 1	20.5 (14.2)
Type 2	10.4 (8.2)
Other or don't know	
Years with diabetes: Median (IQR)	
Type 1	19.5 (9.8‐30.0)
Type 2	8.0 (4.0‐15.0)
Other or don't know	3.5 (20.0‐3.5)

SD, sample standard deviation; IQR, interquartile range.

a These categories are not mutually exclusive.

A vast majority of participants with type 1 or type 2 diabetes (45% and 60%, respectively) reported other health concerns, some of which may be diabetes‐related complications (see Table S1). These concerns were, for types 1 and 2 respectively, eye complications (34% and 15% of participants), heart complications (13% and 24% of participants), kidney complications (22% and 8% of participants), mental health complications (34% and 27% of participants) and nerve complications (40% and 30% of participants). Many participants reported not having been screened for these complications in the previous year. Of those with type 1 and type 2 diabetes, respectively, 63% and 78% reported not receiving screening for eye complications within the past year; 71% and 68% reported not receiving screening for heart complications; 53% and 70% reported not receiving screening for kidney complications; 68% and 86% reported not receiving screening for mental health complications; and 61% and 70% reported not receiving screening for nerve complications.

#### 3.1.2 Focus groups

Of the 23 people who initially expressed an interest in participating in the study, 5 were ineligible because they neither had diabetes nor cared for a person with diabetes; 2 withdrew because they were unavailable on the day of the focus group and 2 withdrew without explanation. Of the 14 remaining individuals who participated in the 3 focus groups, 7 (50%) were female and 3 total (21%) were living with type 1 diabetes. The characteristics of participants are shown in Table 2.

**Table 2:** Focus Groups: Characteristics of the 14 participants

Focus group number	Pseudo	Sex	Treatment	Type of diabetes	Number of years with illness	Age of participants in focus group (mean/range)
1	Part1F4	F	Insulin‐Humalog	1	19	52 [45‐55]
1	Part1F2	F	Metformin‐Glucophage‐Onglyza	2	10	
1	Part1F3	F	Metformin	2	4‐5	
2	Part2F3	F	Multiple injections	1	28	59 [46‐64]
2	Part2F4	F	(Insulin lente) Metformin	2	10	
2	Part2H1	M	Unknown	2	Over 20	
2	Part2H2	M	Janumet and diamicron	2	10	
2	Part2H3	M	Glucophage	2	7‐8	
2	Part2H4	M	Janumet	2	10	
3	Part3H3	M	Unknown	2	8 mo	58 [35‐74]
3	Part3H1	M	Insulin	1	15	
3	Part3H2	M	Unknown	2	11	
3	Part3F2	F	Metformin and glyburide	2	13	
3	Part3F3	F	Metformin	2	15	

### 3.2 Data analysis

#### 3.2.1 Online survey

We report here the medians rather than the means because the distribution of responses to the survey questions about the importance of research topics regarding preventing and treating the complications of diabetes was not symmetrical. The median scores for people with both type 1 and type 2 diabetes were between 84 and 100 (on a 0 to 100 rating scale, with 100 indicating extremely important) indicating that participants assigned high importance to all 10 predetermined research topics with relatively little variation between topics. Topics that had the highest median scores and the least variation in responses were preventing and treating kidney, eye, heart and nerve problems. Research topics for which participants had the widest interquartile range in scores were as follows: preventing and treating mental health problems, developing and testing smart insulin, patient and caregiver education, and artificial pancreas research (type 1) (see Table 3).

**Table 3:** Online survey results regarding the importance of diabetes‐related research topics

	Median importance rating		Illustrative quotes
	Med (IQR)		
Develop and test ways to:	Type 1 diabetes	Type 2 diabetes	
… help people with diabetes prevent and treat kidney problems	100 (IQR 82.0‐100.0)	97 (IQR 82.0‐100.00)	I've had chronic kidney disease for 15 y which eventually led to 3 y of peritoneal dialysis. It is a horrible thing to have to undergo. *22 y old woman living with type 1 diabetes*.Not only are diabetics generally at higher risk of kidney disease, the medication I'm on can make it worse. It's important to be screened to avoid complications. *37 y old woman living with type 2 diabetes*.
… help people with diabetes prevent and treat eye problems	100 (IQR 81.5‐100.0)	97.5 (IQR 81.0‐100.0)	Retaining vision helps independence and quality of life. *34 y old man living with an adult with type 1 diabetes*. I am not aware of as many diseases that can cause blindness as diabetes. If possible to prevent, then many people would not have such a drastic, life changing symptom. They would be able to live the life they had planned/intended. *44 y old woman living with type 2 diabetes*.
… help people with diabetes prevent and treat heart problems	100 (IQR 82.5‐100.0)	93.0 (IQR 81.0‐100.0)	It is hard to control narrowing of the arteries, so if research could help us understand this ‐ it would be wonderful. *35 y old man living with type 1 diabetes*.Very important, cardiac problems often lead to death. My husband suffers from heart disease, he uses a pacemaker defibrillator, his heart only operates at 28%. *58 y old woman living with type 2 diabetes*.
… help people with diabetes prevent nerve problems	99.0 (IQR 76.0‐100.0)	99.0 (IQR 76.0‐100.0)	Doctors need to take this entire disease more seriously. Not simply checking A1C levels once a year. My uncle lost both legs and later passed away of heart problems that were diabetes related. The more active we remain the better our chances at remaining healthy. The ability to use out legs is a huge setback and should not be allowed to occur. *30 y old woman living with type 2 diabetes*.
… help people with diabetes prevent and treat mental health problems	84 (IQR 61.3.0‐100.0)	88.0 (IQR 63.0‐100.0)	I suffer from this presently and feel that it is just the way my life has become. I think it is very important that those who need help with mental health issues, be given help right away and on a regular basis. Diabetes has changed my life and I do not enjoy life as I once did. I just keep getting more health problems on top of diabetes, all things that I have always been afraid of having such as high blood pressure and cholesterol and weight problems. I have gained a lot of weight since being diagnose with diabetes and I was always under the impression that people with diabetes lost a lot of weight. Now losing weight is next to impossible for me. I have tried everything… and also paid a lot of money to lose weight and I just cannot and this is causin me even more health problems. *25 y old woman living with type 1 diabetes*.People need to have testing available because mental health issues affect your entire lifestyle. It is important to be able to find help in handling stress due to diabetes. *42 y old woman living with type 2 diabetes*.
… develop and test an artificial pancreas	92 (IQR 66.0‐100.0)	91.0 (IQR 71.0‐100.0)	If I don't have to worry about adjusting an insulin dose all day, it will be easier to maintain a more natural life style, and possibly a healthier lifestyle. *41 y old woman living with type 2 diabetes*.
… develop and test smart insulin	84.0 (IQR 61.3‐100.0)	93.0 (IQR 81.0‐100.0)	This would represent a miracle in the area of blood sugar management. For an artificial insulin to mimic naturally produced insulin will result in better management of blood sugar levels and reduce dramatically the physical complications associated with them. *43 y old man living with type 1 diabetes*.This would be a boon to older people or persons such as my mother who has macular degeneration and has problems testing and then seeing the results. *48 y old man living with type 2 diabetes*.
… develop and test continuous glucose monitoring	92 (IQR 70.5‐100.0)	88.0 (IQR 64.5‐100.0)	It would be nice to measure glucose levels at the point of origin. Greater accuracy and less pain for the patient. All good. In addition, a greater precision in measurement means greater accuracy in dosing medication. *25 y old man living with an adult with diabetes*.
… develop and test patient and caregiver education	83.0 (IQR 65.8‐100.0)	92.0 (IQR 70.0‐100.0)	I believe self‐management is the most important part of the diabetes care. Doctors and nurses cannot monitor patients 24/7. The patients and the caregivers have to take care of the patients on daily basis. *29 y old woman living with adult with type 1 diabetes*.Essential, because in my experience doctors are more interested in providing treatment and not discussing options or educating the patient, you are essentially left on your own to learn and discover. *44 y old man living with type 2 diabetes*.
… develop and test programs that teach health care professionals	91 (IQR 71.0‐100.0)	85.5 (IQR 77.0‐100.0)	This is very important. I have had experience with health care providers who are very good at applying their chosen healthcare applicable field yet fail to appreciate the little nuances of actually living with the affliction and what this means when making daily choices in consideration of it. *43 y old man living with type 1 diabetes*.This is not a diabetic issue, but a patient care issue. Not that it is not important, but it is broader than just diabetes. *42 y old woman living with type 2 diabetes*.

Cronbach's alphas were .94, .93 and .94, respectively, for the Fear of Complications Scale,^28^ Hypoglycemia Fear Scale^24^, ^25^, ^26^ and Diabetes Distress Scale.^27^ People with type 1 diabetes and type 2 diabetes had mean scores of 23 (SD 10) and 18 (SD 10), respectively, on the Fear of Complications Scale (range 0‐45). Participants with type 1 diabetes had a mean score of 34 (SD 17) on the Hypoglycemia Fear Scale (range 0‐108) indicating sometimes fearing hypoglycaemia, while participants with type 2 diabetes had a mean score of 21 (SD 16) indicating being concerned less often. Finally, participants with type 1 diabetes and type 2 diabetes had mean scores of 2.81 (SD 1.23) and 2.23 (SD 1.27) on the Diabetes Distress Scale. Using the cut‐off score recommended by Fisher et al^38^ this indicates that on average, participants with type 1 diabetes had moderate but non‐clinical levels of distress (threshold = 3) (see Table S1).

Comments provided by participants in the open box sections of the survey aligned with the quantitative findings and illustrate the emotional distress linked to diabetes and diabetes management, the fear associated with episodes of hypoglycaemia and its consequences, and with the long‐term complications of the disease (see Table 3).

#### 3.2.2 Focus groups

The thematic analysis allowed us to identify a set of general concerns about diabetes‐related complications as reported by members of under‐represented groups.

### 3.3 General concerns about diabetes‐related complications

Participants in the focus groups provided further insight into the nature of their concerns about the impact of diabetes on their quality of life, life‐expectancy (Table 4, citation 1) and vulnerability to other diseases (Table 4, citation 2). Most participants reported being most afraid of complications that potentially lead to functional impairment (blindness), additional morbidity (chronic renal failure) or death (hypoglycaemia) (Table 4, citation 3). Furthermore, participants pointed to the challenge of continuously monitoring and managing the disease (Table 4, citations 4, 5 and 6).

**Table 4:** Citations from focus groups participants

Citation	Participant ID
*Theme 1: General concerns about diabetes‐related complications*	
Citation 1: The problem with … any disease that it may, uh … it will get worse if you have diabetes. That's my greatest fear. Ok. my mother is 99 y old, next year she will be 100. And last year, they took her to the hospital and because of her age, we thought: it's over. After a week with the medication, the doctor said, “She can go home.”. And, we say: “What do you mean she can go home? “. Yes, because she is not sick, and there are no complications. We gave her the standard treatment for pneumonia and it's gone. But my fear is that anything we catch, those of us who have diabetes, it can complicate things. It is more serious compared to those who don't have diabetes.	F3FG3T2D
Citation 2: Those of us with diabetes are very vulnerable. We can have, have any number of diseases, our organs are not properly supplied with blood sugar … so if we wait too long to eat, uh … there is damage. We start, for example, to experience symptoms such as arthritis … … circulation problems and the worst is … that once the onset begins, once we have arthritis … is too late […]. We can't go back and say: “Treat it. “ So the fear is […] we're on a tightrope. We never know when we might develop it.	H2FG3T1D
Citation 3: I think that my two main concerns [about diabetes] are … eye and kidney problems. We can live if you lose a few toes … but if one of my kidneys is removed, I will have just one left. I don't have 10 of them like my fingers. Same with the eyes … becoming blind. That means, forget your car … find a way to shop. Park your car… So that's what bothers me the most.	F4FG2T2D
Citation 4: […] it's been 28 y since I've had to prick my fingers. The pump, I wanted it, but I had still had to prick my fingers. But as I don't like to be tightened, I thought: “What will the pump add if I still have to prick my fingers? I think that the day there is a way to […], use a small injection … right there … to know your blood sugar, not just capillary, […] … not just intercellular but really uh … uh … like when you prick your fingers. Oh, I'll be happier then. That's the only thing that really wears me out […].	F3FG2T1D
Citation 5: Once, my skin was hard as leather. I had trouble injecting myself. So I stopped.	F3FG2T2D
Citation 6: I type, I am secretary, imagine. So … I can`t be blasting people. [Laughs] I can't wait for them to invent a machine. They have invented insulin pumps, stuff like that, but will they end up inventing a machine for us to take our blood sugar without always needing to prick our fingers?	F4FG2T1D
Citation 7: And uh … well it's perhaps not related, but I will mention it […] me my mother had diabetes. And uh … I took her to the hospital in a diabetic coma. So I have always been scared of developing a … diabetic coma. So sometimes I ate more, when I worked out, because I was afraid of falling. So I did not pay close attention to my blood sugar, to take it … so sometimes … I raised my blood sugar to ensure that it would not fall too low while I was exercising.	F3FG1T2D
	F3FG1T2D
Citation 8: Well … I talked to the doctor I saw. And … so I understood that I could go down to 4.6, without fainting like my mother. But I am still traumatized by that. When I put my mom in an ambulance, I was … mom. So I am still affected by it … its something I fear.	
Citation 9: Is your research program going to look at the cost of medication […] cause its important […] for patients to be able to voice their opinion on the matter […]	H3FG2T2D
*Theme 2: Need to better understand the danger of polymedication toxicity in patients with multiple comorbidities*	
Citation 10: Is there a research program involving people who take antipsychotics … and its effects […] I consulted a mental health organisation and … a lot of us have marginal blood sugar levels […] Those of us who take antipsychotics …[have marginal blood sugar levels]	H2FG2T2D
*Theme 3: Need to better understand barriers to quality care for immigrants living with diabetes*	
Citation 11: As the lady said, here you really need to knock … ask …here people are used to knocking on doors and requesting help when there is a problem. We come from a country where you do not make demands. Instead, we'll go and we'll say, “I feel bad.” And then we wait for the nurse or doctor to say, “Okay, come on in. “. So the system [here] is very good except that there is a lack of awareness, especially you are an immigrant. We are treated differently, we are not treated like other Canadians, especially we don't speak the language well. PartH2FG3T1D	H2FG3T1D
Citation 12: […] When I got here. I've been here for about a year. […] I went to ask [what] I needed to buy insulin […]. The doctor said, “Go to the pharmacy. Talk to the pharmacist about what insulin you need. “. I said, “what is that? You're the doctor. It is not [a pharmacist who should tell me what insulin I need]. “ But, he said. “Ok Go ahead. “. I can do anything. So I thought: “Oh … is how they practice medicine here? […] So now … I'm afraid to go to the doctor because I do not know if he's able to treat me. […]. I don't know how it works exactly […].	H3FG3T2D
Citation 13: In my case, I was diagnosed, the diagnosis was in 2004 but I already had symptoms. Maybe even 10, 15 y before that. And the problem is that the doctors I saw did not know that in Latin America there is a high rate of diabetes. They never gave me the right medication. So for 10 y or so I had symptoms of diabetes but I did not know what it was.	H2FG3T1D
*Theme 4: Better dissemination of the results of research on diabetes*	
Citation 14: Because me if I … me, my problem, when they took me here … last year. I was a bit like you. When I went to do physical activity. I ate like two snacks before noon. […], one at 10:00, and then another, I'd say, “Well, I'll take one at 11:00. “. But that was what I learned… 20 y earlier. But, [the knowledge] has evolved, changed. So, I did not have to do that anymore. […], so I relearned to live with diabetes.	F4FG1T1D
Citation 15: Given that my pancreas, the reserves … And I would have liked someone to explain me how it worked, a little, because I could have taken care of myself, but I did not. So now, my reserves are gone. … I Can't catch up anymore. It's not possible. That's what my doctor said.	H2FG2T2D
Citation 16: I went through a divorce with it [diabetes], probably because I was difficult, you know me I'm diabetic. I can't eat just anything, I always had to bring it up […]. And in the family, and we were a large family, I was the only diabetic, but people didn't understand. Yeah, take it there, it does not matter. They would say take this, it won't hurt. I tried to explain. One is not a prophet in one's own land huh. That's for sure.	F3FG2T1D
Citation 17: And them [my children and my spouse], they not believe me when I got down to 4.6 and I told them that I had to eat. “Oh mom, stop with your diabetes”. But my head would be spinning. And they're like “mom, stop with your diabetes”. I said, “I can't wait”. […] Then with my partner when we go walking […]. And at a moment, I feel … I call it spinning, dizzy. Then I would say “stop I have to eat”. [And he'd say] Well there you go, see how big you are, you eat all the time.	F3FG1T2D
Citation 18: Then, it made my children anxious. It makes them anxious. The fact that I had diabetes, […] since I had hypoglycemia quite often […] my voice would change. So they knew … […] … but I felt like … uh … I was affecting them. Because […] they already had a ADHD [attention deficit hyperactivity disorder] problem. That this didn't help either.	F3FG2T1D

In addition to these general and common concerns, four specific themes arose from the focus group discussions:

### Theme 1: The bidirectional relation between individual history and socio‐economic context, and the management of diabetes

Two aspects of individual history and context were mentioned by participants: (i) the influence of previous life‐experiences on the management of diabetes; and (ii) the impact of socio‐economic conditions on the outcomes of the disease. Regarding the first point, participants said they suspected a strong relation between their previous life‐experiences and the management of diabetes‐related complications. They wished that this relation could be investigated. For example, one participant talked about adopting bad eating habits such as dieting during the day and binging at night because she saw a loved one in a diabetic coma. (Table 4, citations 7 and 8).

With respect to the second point, discussions in both focus groups focused on the need for studies examining the cost of diabetes treatment (Table 4, citation 9). For example, some participants argued that they sometimes had to choose between paying their rent and buying insulin and complained that this should be a concern to researchers.

### Theme 2: The need to better understand the danger of polymedication toxicity in patients with multiple comorbidities

Focus group participants who were either elderly or had experienced mental health problems expressed their concerns about toxic drug interactions resulting from polymedication. They stressed the urgent need to understand, whether and/or to what extent, there may be interactions between their diabetes medication and other treatments (Table 4, citation 10).

### Theme 3: The need to better understand barriers to quality care for immigrants living with diabetes

Focus group participants who were immigrants had two core concerns regarding diabetes and its related complications for researchers to address, notably: (i) how to improve access to quality care for immigrants with diabetes; and (ii) how to make health‐care professionals more knowledgeable about the specific care needs of immigrants living with diabetes. Most immigrants in the study talked about cultural or linguistic barriers to navigating the health system. For example, one participant talked about how she had learned to be assertive in expressing her needs (Table 4, citation 11). Another participant talked about his experience going back and forth between the doctor and the pharmacist without answers to his needs (Table 4, citation 12). Finally, participants who were immigrants unanimously reported that health‐care professionals were inadequately trained to detect symptoms and diagnose diabetes among individuals who are newcomers to the country. One participant, for example, said that this led to a failure to recognise pre‐diabetes symptoms, forcing this person to consult multiple physicians before a glycaemia test was requested (Table 4, citation 13).

### Theme 4: The need for better dissemination of the research results on diabetes

Focus group participants also expressed concerns about not having access to updated information on diabetes. They reported being aware of on‐going research, but were never informed by community organizations about the research results (Table 4, citations 14 and 15).

Participants also pointed to a need for better information for their loved ones and relatives, to help them understand and provide better support in the management of the disease (Table 4, citations 16, 17, 18).

## 4 DISCUSSION

This study aimed to explore the importance of diabetes‐related complication research topics relevant to those living with or caring for someone living with diabetes. Additionally, we wished to explore the reasons why these topics are important from the perspective of under‐represented populations. Findings from both the quantitative and qualitative components of the study complement each other and can be summarized in three main points.

First, the alignment of what is important for patients in diabetes research. Both survey and focus group participants indicated the importance of preventing and treating well‐known complications of diabetes such as kidney, eye and nerve problems. This finding confirms that research on such complications matters to patients and caregivers. Second, the need for more research about the bidirectional influence of the “life context” on diabetes.*** ***Our participants also pointed out that there are a number of individual and contextual factors, such as individual circumstances (eg, life conditions, previous experiences), socio‐economic status and the experience of managing the condition that need further exploration, especially for the most under‐represented people included in this study. Finally, the third point was the need to deepen diabetes‐related research in under‐represented populations. Our results further suggest that research topics should be tailored to address specific challenges such as access to culturally relevant care for immigrants.^39^

Consistent with other studies,^40^, ^41^ our quantitative data show that participants had moderate levels of emotional distress around diabetes‐related complications. Our qualitative analysis provided some insight into the nature of these concerns. For example, the fear that diabetes‐related complications (eg, kidney failure or blindness) may result in functional impairment or death (eg, as a result of a hypoglycaemia). Additionally, fears were often amplified not only by personal experience as shown in other studies,^42^, ^43^, ^44^, ^45^, ^46^ but also by witnessing others dealing with such complications (such as having seen a loved one with kidney failure or experiencing a hypoglycaemic episode). These experiences impact how research topics are rated by those whose lives are touched by the disease. Unfortunately, further investigation of these questions was not possible with this study for two reasons: (i) the focus group participants were not asked to rate the complications as did online survey participants; and (ii) the focus groups were conducted separately from the quantitative portion of the study.

Overall, our findings point to a need for more research on diabetes, its complications and the bidirectional influence of a number of individual and contextual factors such as individual circumstances (eg, life conditions, previous experiences, emotional distress); socio‐economic status; and the experience of managing the condition, especially for the most under‐represented groups included in this study. It was suggested that research topics should be tailored to address specific challenges, such as access to culturally competent care for immigrants.^39^

Our study did, however, have a few limitations. Due to time and budget constraints, focus group activities were restricted to Montreal and Quebec City, where our team is based. This limited our ability to recruit in other cities across Canada and also limited a true representation of the country's population. Although our respondents and their experiences reflect a broad sample of the population of Canada, several other groups who may have particular needs (eg, pregnant women, Indigenous peoples, parents or guardians of children with diabetes, as well as caregivers) were under‐represented in the online survey and were absent in the focus groups. Therefore, our sample lacks representation of some other under‐represented populations in Canada. Additionally, language barriers may have limited our selection of participants and excluded individuals, particularly those from under‐represented groups such as immigrants. Furthermore, because this online survey and focus group based study relied on participant self‐reports, the data could be limited by the subjects’ ability for introspection, their individual interpretations and social desirability bias.^47^ Finally, because this was a preliminary study aimed at exploring the importance of different research topics to those living with diabetes and caregivers in Canada, we did not undertake prioritization activities that require trading‐off one priority against another to produce a ranked list. Such activities are planned for future research.

One strength of this study is its use of qualitative and quantitative methods to help capture the experiences of under‐represented groups and diverse participants from across Canada. This approach proved feasible as a method for efficiently exploring patients’ and caregivers’ preliminary views on research topics within a short period of time.

## 5 CONCLUSIONS

This study confirmed the importance of research topics regarding diabetes‐related complications within a population of people living with diabetes or caring for someone with diabetes, and further explored reasons why these topics might be important for certain groups of under‐represented people. The results of this study about what matters most to people living with, and caring for those living with diabetes, including people from under‐represented populations, informed the research program of a 5‐year pan‐Canadian Strategy for Patient‐Oriented Research Network on Diabetes and its related complications (2016‐2021).^39^ A broad range of people living with diabetes are now involved as patient partners in this network, collaborating on research projects, research planning and supporting network governance. We anticipate that our results and on‐going work will contribute to the development of targeted interventions better aligned with improving the health and well‐being of people whose lives are touched by diabetes.

## AUTHOR CONTRIBUTIONS

M.J.D. provided the study concept and design, supervised the protocol development and research, enrolled patients for the qualitative stage, facilitated focus groups, analysed data and provided the first draft of the manuscript. S.C.D. conducted the descriptive statistics, wrote the quantitative part of the manuscript, reviewed and edited the manuscript. M.T.D. enrolled patients for the qualitative stage, facilitated focus groups, analysed data and wrote the manuscript. F.L. reviewed and edited the manuscript. H.O.W. supervised the survey data collection, reviewed and edited the manuscript. M.J.D. is the guarantor of this work and, as such, had full access to all the data in the study and takes responsibility for the integrity of the data and the accuracy of the data analysis.

## CONFLICT OF INTEREST

The authors report no conflict of interest.
