# Supplementary material for: Diabetes‐related complications: Which research topics matter to diverse patients and caregivers?
Source: Health Expect. 2017 Nov 22;21(2):549–59. doi: 10.1111/hex.12649 (PMC5867328; doi:10.1111/hex.12649)
Supplement: Supplementary file 1 [file HEX-21-549-s001.docx]

Supplemental Questionnaire 1: Survey about diabetes-related problems

**Title/Header: Welcome**

Thank you for your time.

**Background on the project:**

This study is being led by Dr. Joyce Dogba, MD, PhD, and Dr. Holly Witteman, PhD, who are researchers and professors at the medical school of Laval University in Quebec City, Canada. Dr. Witteman is also a person who has lived with type 1 diabetes since childhood.

By participating in this study, you will help doctors and scientists find ways to better prevent or treat complications of diabetes.

**Who can participate:**

To take this survey, you must be 18 years old or older, live in Canada, and either have diabetes yourself or care for a child or adult with diabetes.

**What your participation entails:**

We will ask you to answer some questions about your opinions and feelings about diabetes-related problems. We will also ask you to answer a few questions about yourself.

Answering these questions will take about 10−20 minutes.

Your answers will be used to help a team of doctors and scientists in Canada decide on their research topics while paying attention to problems that matter to people with diabetes, their families and loved ones.

**Confidentiality:**

Your name will not be recorded anywhere in this survey. All your answers will be kept confidential. We may quote your answers in conference presentations or scientific articles but we will not identify you personally.

Please do not provide identifying information or names of health care professionals in this survey. (In other words, don’t give doctors’ names or names of other people on your health care team.) If you accidentally provide identifying information, we will remove it before we do any analysis.

At the end of this survey, we will ask you if you are willing to be contacted to participate in a discussion group of people with diabetes or families of people with diabetes. If you provide contact information, we will store it separately from your other answers to make sure your answers stay anonymous.

**Consent:**

By completing the online survey you are consenting to participate in the study. You can stop the survey at any time and if you don’t finish, we will assume that you don’t want to participate in the study. In that case, we will not keep or use any of the information you have provided.

You may skip any questions you don’t want to answer.

**Sharing the results:**

All the information gathered in this study will be used for **research and education purposes only** and will never be shared for any other purpose.

**Questions:**

This study was approved by the Research Ethics Committee at the CHU de Québec. If you have any questions about the study you can contact the principal investigators:

M. Joyce Dogba or Holly O. Witteman

Faculté de Médecine

Université Laval

1050, rue de la Médecine

Telephone: 418 656-2131, extension 3981 (Witteman) or 2235 (Dogba)

email: Joyce-Maman.Dogba@fmed.ulaval.ca or Holly.Witteman@fmed.ulaval.ca

**Concerns or complaints:**

If you have any concerns or complaints please contact the CHU de Québec’s Ombudsman’s office at: Telephone: 418-654-2211

**Please feel free to print this sheet for your records.**

**Your help means a lot to us. We thank you for taking the time to participate in this study.**

**Title/Header: Your connection to diabetes**

**To participate in this study, you must (1) be 18 years or older, (2) live in Canada, and (3a) currently have diabetes yourself or (3b) currently take care of someone with diabetes.**

1. What is your age? I am _______ years old.
2. Do you live in Canada? (choose one)
   1. Yes
   2. No
3. What is your relationship to diabetes? (check all that apply)
   1. I have diabetes myself
   2. I am a parent of a child who has diabetes
   3. I care for an adult with diabetes
   4. None of the above

**Title/Header: About your diabetes**

*N.B.: In this appendix we present the questions for people who indicated that they themselves had diabetes. Similar questions were asked of caregivers, with wording variations as required to refer their child or loved one.*

1. Which type of diabetes do you have? (choose one)
2. type 1
3. type 2
4. I don’t know
5. other type ____________

optional comments: [comment box]

1. How long have you had diabetes? ____________ years OR what year were you diagnosed with diabetes? _______________

optional comments: [comment box]

1. Do you **currently use** any of the following **diabetes technology or medication** in this **alphabetical list**? (check all that apply)
2. Artificial pancreas (an insulin pump with a continuous glucose monitor that makes decisions on its own)
3. Continuous glucose monitor (this usually requires wearing a sensor, may be stand alone or with a pump)
4. Home blood test meter and test strips (this usually requires pricking a finger)
5. Insulin (any kind), injected by needle or pen
6. Insulin pump (any brand)
7. Nightscout, Dexcom Share, xDrip, or other cloud-based system
8. Other medication you give by needle (e.g., Byetta, Symlin, Victoza, etc.)
9. Other _______________________
10. Pills for diabetes

**Title/Header: About you**

Remember, all questions in this survey are optional. The more information you provide, the more helpful it is to us, but you do not have to answer any questions that make you uncomfortable.

1. Do you identify as: (check one)
2. Female
3. Male
4. Other ___________________
5. I prefer not to say
6. Do you identify as: (check all that apply)
7. Aboriginal (First Nations, Métis, Inuit)
8. Asian (including South Asian)
9. Black
10. Hispanic
11. Middle Eastern
12. White or Caucasian
13. Other __________________
14. I prefer not to say
15. What is the highest level of education you have completed? (check one)
16. None
17. Elementary school
18. High school
19. Trade school
20. Some postsecondary education, but no degree
21. Associate’s degree, college diploma, or equivalent (AA, AS, CEGEP, etc.)
22. Bachelor’s degree (BA, BSc, etc.)
23. Graduate or professional degree (MA, MPH, PhD, MD, etc.)
24. I prefer not to say
25. Where do you live in Canada? (check one)
26. Alberta
27. British Columbia
28. Manitoba
29. New Brunswick
30. Newfoundland/Labrador
31. Northwest Territories
32. Nova Scotia
33. Nunavut
34. Ontario
35. Prince Edward Island
36. Quebec
37. Saskatchewan
38. Yukon
39. Other __________________
40. I prefer not to say
41. Were you born (check one)
42. in Canada
43. outside Canada
44. I prefer not to say
45. What is your total household income per year before tax? Household income means all income of everyone living with you. (check one)
46. Less than $20,000 per year
47. $20,000 to $39,999 per year
48. $40,000 to $59,999 per year
49. $60,000 to $79,999 per year
50. $80,000 to $99,999 per year
51. $100,000 or more per year
52. I prefer not to say

**Title/Header: Your experiences with diabetes-related problems**

Now we are going to ask you some questions about your health. Remember, answering this survey is optional. Your answers will help scientists in Canada make sure they are working on things that matter to people with diabetes.

1. Have you **ever had**: (check one answer for each)
2. Eye problems related to diabetes (e.g., diabetic retinopathy) [yes, no, I don’t know]
3. Heart problems (e.g., heart disease, heart attack, stroke) [yes, no, I don’t know]
4. Kidney problems (e.g., chronic kidney disease, renal disease) [yes, no, I don’t know]
5. Mental health problems (e.g., depression, anxiety) [yes, no, I don’t know]
6. Nerve problems (e.g., numbness, tingling, chronic pain or loss of feeling in your feet, legs, or elsewhere) [yes, no, I don’t know]
7. Other diabetes-related problems [yes, no, I don’t know] (please specify) _________________
8. In **the past year**, have you had tests done to check for: (check one answer for each)
9. Eye problems related to diabetes (e.g., diabetic retinopathy) [yes, no, I don’t know]
10. Heart problems (e.g., heart disease, heart attack, stroke) [yes, no, I don’t know]
11. Kidney problems (e.g., chronic kidney disease, renal disease) [yes, no, I don’t know]
12. Mental health problems (e.g., depression, anxiety) [yes, no, I don’t know]
13. Nerve problems (e.g., numbness, tingling, chronic pain or loss of feeling in your feet, legs, or elsewhere) [yes, no, I don’t know]
14. Other diabetes-related problems [yes, no, I don’t know] (please specify) _________________
15. Do you have any other health challenges besides diabetes and anything you checked above? For example, you may also have arthritis, chronic obstructive pulmonary disorder (COPD), eczema, allergies, or something else. (choose one)
16. Yes (please specify) ______________
17. No
18. I don’t know
19. Feel free to add any comments about your health that you would like to share with us. [open comment box]

**Title/Header: Your feelings about diabetes-related problems, technology, and education**

Now we would like to ask you some questions about diabetes-related problems, technology, and education. Remember, we are interested in **your honest feelings and opinions**.

Some questions may feel repetitive. We ask questions that are similar to each other because it helps us make sure we truly understand everyone’s opinions.

*N.B.: The following 10 questions were presented in random order to avoid order effects.*

1. People with diabetes are at higher risk of **kidney problems**. People who have kidney problems may need to have dialysis, which means being hooked up to a machine that cleans your blood at least several times a week. Kidney problems may also lead to death.

   In your opinion, how important is it for doctors and scientists to **develop and test ways to** help people with diabetes **prevent and treat** **kidney problems**? [slider anchored at “not important at all” on left, “extremely important” on right]

Can you briefly explain your answer? In your opinion, why is this important or not important? [open text]

1. People with diabetes are at higher risk of **eye problems**. People who have eye problems related to diabetes (often called ‘diabetic retinopathy’) can lose their vision (meaning they may become blind.)

In your opinion, how important is it for doctors and scientists to **develop and test ways to** help people with diabetes **prevent and treat** **eye problems**? [slider anchored at “not important at all” on left, “extremely important” on right]

Can you briefly explain your answer? In your opinion, why is this important or not important? [open text]

1. People with diabetes are at higher risk of **heart problems**. People who have heart problems may have a heart attack or stroke, which could lead to death.

In your opinion, how important is it for doctors and scientists to **develop and test ways to** help people with diabetes **prevent and treat** **heart problems**? [slider anchored at “not important at all” on left, “extremely important” on right]

Can you briefly explain your answer? In your opinion, why is this important or not important? [open text]

1. People with diabetes are at higher risk of **nerve problems**. People who have nerve problems may have problems like pain, tingling or numbness, usually in their feet. Having these kinds of nerve problems increases the risk of needing to have amputation. Amputation means having a body part cut off.

In your opinion, how important is it for doctors and scientists to **develop and test ways to** help people with diabetes **prevent and treat** **nerve problems**? [slider anchored at “not important at all” on left, “extremely important” on right]

Can you briefly explain your answer? In your opinion, why is this important or not important? [open text]

1. People with diabetes are at higher risk of **mental health problems**. People who have mental health problems may have problems like depression, anxiety, eating disorders, or other mental health problems. Such problems may have to do with the stress of living with diabetes. Mental health problems can make it more difficult for people to manage their diabetes.

In your opinion, how important is it for doctors and scientists to **develop and test ways to** help people with diabetes **prevent and treat** **mental health problems**? [slider anchored at “not important at all” on left, “extremely important” on right]

Can you briefly explain your answer? In your opinion, why is this important or not important? [open text]

1. People with diabetes who are taking insulin may benefit from an **artificial pancreas**. An artificial pancreas is a system that combines an insulin pump with a sensor that continuously measures glucose (sugar). The pump contains insulin (to lower blood sugar) and may also contain glucagon (to raise blood sugar). The system uses the sensor to decide how much insulin or glucagon the person needs at that time.

In your opinion, how important is it for doctors and scientists to **develop and test an artificial pancreas**? [slider anchored at “not important at all” on left, “extremely important” on right]

Can you briefly explain your answer? In your opinion, why is this important or not important? [open text]

1. People with diabetes who are taking insulin may benefit from **smart insulin**. Smart insulin is insulin that automatically adjusts to the glucose (sugar) in people’s blood. With smart insulin, people with diabetes could take just one injection a day, and the insulin would “turn on” whenever it was needed and “turn off” when not needed.

In your opinion, how important is it for doctors and scientists to **develop and test smart insulin**? [slider anchored at “not important at all” on left, “extremely important” on right]

Can you briefly explain your answer? In your opinion, why is this important or not important? [open text]

1. People with diabetes who are taking insulin may benefit from **continuous glucose monitoring**. Continuous glucose monitors are machines that measure glucose (sugar) in the fluid between cells inside a person’s body, using a small sensor inserted under the skin. The machine uses the glucose (sugar) in the fluid between cells to calculate the person’s blood sugar.

In your opinion, how important is it for doctors and scientists to **develop and test continuous glucose monitors**? [slider anchored at “not important at all” on left, “extremely important” on right]

Can you briefly explain your answer? In your opinion, why is this important or not important? [open text]

1. People with diabetes may benefit from **patient and caregiver education and self-management programs**. These are programs that help people learn ways to live well with diabetes and avoid becoming overwhelmed by diabetes, including eating well, being physically active, managing blood sugar, and managing stress.

In your opinion, how important is it for doctors and scientists to **develop and test patient and caregiver education and self-management programs**? [slider anchored at “not important at all” on left, “extremely important” on right]

Can you briefly explain your answer? In your opinion, why is this important or not important? [open text]

1. People with diabetes may benefit from **programs that teach health care professionals how to provide patient-centred care to people with diabetes**. These are programs that help doctors, nurses, dietitians and other health care providers learn techniques that help them listen to patients and focus on what matters to patients.

In your opinion, how important is it for doctors and scientists to **develop and test programs that teach health care professionals how to provide patient-centred care to people with diabetes**? [slider anchored at “not important at all” on left, “extremely important” on right]

Can you briefly explain your answer? In your opinion, why is this important or not important? [open text]

1. Are there **any other issues besides the ones above** that affect people living with diabetes that you feel need more attention from health care professionals and scientists? [open text]
2. Are there **any scientific or other advancements in diabetes** that you think deserve more attention? [open text]

**Title/Header: Last questions**

We would like to ask you a set of questions, divided into three groups. These questions were designed for people with diabetes. If you don’t have diabetes yourself, the questions may still apply to you if you take care of someone with diabetes.

**Title/Header: Distress related to your diabetes**

1. Fear of complications

Please answer each question by selecting the response most appropriate to yourself.

|  |  |  |  |  |
| --- | --- | --- | --- | --- |
| I feel afraid of long-term complications of diabetes (1) | - Very | - Moderately | - A little | - Not at all |
| I worry about losing my eyesight because of diabetes (2) | - All the time | - Frequently | - Occasionally | - Never |
| I worry that having diabetes increases my chances of heart disease (3) | - All the time | - Frequently | - Occasionally | - Never |
| I am afraid I will need a kidney transplant one day (4) | - Very | - Moderately | - A little | - Not at all |
| I am afraid of developing long-term complications as a result of frequent high blood sugars (5) | - All the time | - Frequently | - Occasionally | - Never |
| I am afraid that I may need kidney dialysis one day (6) | - Never | - Occasionally | - Frequently | - Constantly |
| I am afraid that I will develop kidney problems one day (7) | - All the time | - Frequently | - Occasionally | - Never |
| How often do you think about long-term complications of diabetes (8) | - Hardly ever | - Occasionally | - Frequently | - All the time |
| I worry that I might be at a higher risk for having a stroke (9) | - All the time | - Frequently | - Occasionally | - Never |
| Do you ever worry about your future health? (10) | - Not at all | - Occasionally | - Frequently | - All the time |
| I worry that the diabetes specialist will find something wrong with my eyes (11) | - Not at all | - Occasionally | - Frequently | - Constantly |
| Do you ever worry about future problems when your blood sugars are erratic? (12) | - Not at all | - Occasionally | - Frequently | - All the time |
| I am scared that diabetes could affect my feet (13) | - Very | - Moderately | - A little | - Not at all |
| I'm scared of having a heart attack in the future (14) | - Not at all | - A little scared | - Moderately scared | - Very scared |
| I worry about developing problems with circulation (15) | - Never | - Occasionally | - Frequently | - All the time |

1. Fear of Hypogylcemia

Below is a list of things people with diabetes sometimes do in order to avoid low blood sugar and its consequences. Select one of the responses in the drop down menu that best describes What you do in your daily routine to AVOID low blood sugar and its consequences. (Please do not skip any!) To avoid low blood sugar and how it affects me, I ...

|  | Never | Rarely | Sometimes | Often | Very often |
| --- | --- | --- | --- | --- | --- |
| Eat large snacks at bedtime (1) |  |  |  |  |  |
| Avoid being alone when my sugar is likely to be low (2) |  |  |  |  |  |
| Run my blood sugar a little high to be on the safe side (3) |  |  |  |  |  |
| Keep my sugar higher when I will be alone for a while (4) |  |  |  |  |  |
| Eat something as soon as I feel the first sign of low blood sugar (5) |  |  |  |  |  |
| Reduce my medication (insulin/pills) when I think my sugar is too low (6) |  |  |  |  |  |
| Keep my blood sugar higher when I plan to be in a long meeting or at a party (7) |  |  |  |  |  |
| Carry fast-acting sugar with me (8) |  |  |  |  |  |
| Avoid a lot of exercise when I think my sugar is low (9) |  |  |  |  |  |
| Check my sugar often when I plan to in a long meeting or go out to a party (10) |  |  |  |  |  |

Below is a list of concerns people with diabetes sometimes have about low blood sugar. Please read each item carefully (do not skip any). Select one of the responses in the drop down menu that best describes how often you worry about each item because of low blood sugar.

|  | Never | Rarely | Sometimes | Often | Very often |
| --- | --- | --- | --- | --- | --- |
| Not recognizing when I am having low blood sugar (1) |  |  |  |  |  |
| Not having food, fruit, or juice with me (2) |  |  |  |  |  |
| Feeling dizzy or passing out in public (3) |  |  |  |  |  |
| Having low blood sugar while asleep (4) |  |  |  |  |  |
| Embarrassing myself or my friends/family in a social situation (5) |  |  |  |  |  |
| Having low blood sugar while alone (6) |  |  |  |  |  |
| Appearing stupid or drunk (7) |  |  |  |  |  |
| Losing control (8) |  |  |  |  |  |
| No one being around to help me during a low blood sugar (9) |  |  |  |  |  |
| Having low blood sugar while driving (10) |  |  |  |  |  |
| Making a mistake or having an accident at work (11) |  |  |  |  |  |
| Getting a bad evaluation at work because of something that happens when my sugar is low (12) |  |  |  |  |  |
| Having seizures or convulsions (13) |  |  |  |  |  |
| Difficulty thinking clearly when responsible for others (children, elderly, etc.) (14) |  |  |  |  |  |
| Developing long-term complications from frequent low blood sugar (15) |  |  |  |  |  |
| Feeling light-headed or faint (16) |  |  |  |  |  |
| Having low blood sugar (17) |  |  |  |  |  |

1. Diabetes Distress Scale

Living with diabetes can sometimes be tough. There may be many problems and hassles concerning diabetes and they can vary greatly in severity. Problems may range from minor hassles to major life difficulties. Listed below are 17 potential problems that people with diabetes may experience. Consider the degree to which each of the items may have distressed or bothered you, your child or your loved one during the past month and select the appropriate answer.

Please note that we are asking you to indicate the degree to which each item may be bothering you in your life, NOT whether the item is merely true for you. If you feel that a particular item is not a bother or a problem for you, you would select «1». If it is very bothersome to you, you might select «6».

| Feeling that diabetes is taking up too much of my mental and physical energy every day (1) |  |  |  |  |  |  |  |
| --- | --- | --- | --- | --- | --- | --- | --- |
| Feeling that my doctor doesn't know enough about diabetes and diabetes care (2) |  |  |  |  |  |  |  |
| Feeling angry, scared and/or depressed when I think about living with diabetes (3) |  |  |  |  |  |  |  |
| Feeling that my doctor doesn't give me clear enough directions on how to manage my diabetes (4) |  |  |  |  |  |  |  |
| Feeling that I am not testing my blood sugars frequently enough (5) |  |  |  |  |  |  |  |
| Feeling that I am often failing with my diabetes regimen (6) |  |  |  |  |  |  |  |
| Feeling that my friends or family are not supportive enough of my self-care efforts (e.g. planning activities that conflict with my schedule, encouraging me to eat the «wrong» foods). (7) |  |  |  |  |  |  |  |
| Feeling that diabetes controls my life (8) |  |  |  |  |  |  |  |
| Feeling that my doctor doesn't take my concerns seriously enough (9) |  |  |  |  |  |  |  |
| Not feeling confident in my day-to-day ability to manage diabetes (10) |  |  |  |  |  |  |  |
| Feeling that I will end up with serious long-term complications, no matter what I do (11) |  |  |  |  |  |  |  |
| Feeling that I am not sticking closely enough to a good meal plan (12) |  |  |  |  |  |  |  |
| Feeling that my friends or family don't appreciate how difficult living with diabetes can be (13) |  |  |  |  |  |  |  |
| Feeling overwhelmed by the demands of living with diabetes (14) |  |  |  |  |  |  |  |
| Feeling that I don't have a doctor who I can see regularly about my diabetes (15) |  |  |  |  |  |  |  |
| Not feeling motivated to keep up my diabetes self-management (16) |  |  |  |  |  |  |  |
| Feeling that my friends or family don't give me the emotional support that I would like (17) |  |  |  |  |  |  |  |

1. Thank you so much for taking the time to answer these questions. We hope this survey will help doctors and scientists work on things that matter to people with diabetes and the families of people with diabetes. Is there anything else you’d like to tell us?

Supplemental Table 1: Health characteristics of participants who have diabetes (N=397)

|  |  | Type 1 (n=38) | Type 2 (n=354) | Don’t know (n=4) | Other (n=1) |
| --- | --- | --- | --- | --- | --- |
|  | | | | | |
| Has other health concerns besides diabetes: n (%) | Yes | 17 (44.7) | 213 (60.2) | 4 (100) | 1 (100) |
|  | No | 20 (52.6) | 131 (37.0) | 0 | 0 |
|  | I don’t know | 0 | 6 (1.7) | 0 | 0 |
|  | Not reported | 1 (2.7) | 4 (1.1) | 0 | 0 |
|  | | | | | |
| Has eye problems: n (%) | Yes | 13 (34.2) | 54 (15.2) | 0 | 1 (100) |
|  | No | 24 (63.2) | 276 (78.0) | 3 (75.0) | 0 |
|  | I don’t know | 0 | 24 (6.8) | 1 (25.0) | 0 |
|  | Not reported | 1 (2.6) | 0 | 0 | 0 |
|  | | | | | |
| Has been screened for eye problems in the past year: n (%) | Yes | 24 (63.2) | 206 (58.2) | 2 (50.0) | 0 |
|  | No | 13 (34.2) | 141 (39.8) | 1 (25.0) | 1 (100) |
|  | I don’t know | 0 | 7 (2.0) | 1 (25.0) | 0 |
|  | Not reported | 1 (2.6) | 0 | 0 | 0 |
|  | | | | | |
| Has heart problems: n (%) | Yes | 5 (13.2) | 83 (23.4) | 1 (25.0) | 0 |
|  | No | 32 (84.2) | 263 (74.3) | 3 (75.0) | 1 (100) |
|  | I don’t know | 0 | 7 (2.0) | 0 | 0 |
|  | Not reported | 1 (2.6) | 1 (0.3) | 0 | 0 |
|  | | | | | |
| Has been screened for heart problems in the past year: n (%) | Yes | 11 (28.9) | 111 (31.3) | 1 (25.0) | 0 |
|  | No | 27 (71.1) | 241 (68.1) | 2 (50.0) | 1 (100) |
|  | I don’t know | 0 | 2 (0.6) | 1 (25.0) | 0 |
|  | Not reported | 0 | 0 | 0 | 0 |
|  | | | | | |
| Has kidney problems: n (%) | Yes | 8 (21.0) | 29 (8.2) | 1 (25.0) | 0 |
|  | No | 26 (68.4) | 314 (88.7) | 3 (75.0) | 1 (100) |
|  | I don’t know | 2 (5.3) | 8 (2.3) | 0 | 0 |
|  | Not reported | 2 (5.3) | 3 (0.8) | 0 | 0 |
|  | | | | | |
| Has been screened for kidney problems in the past year: n (%) | Yes | 18 (47.4) | 104 (29.4) | 1 (25.0) | 0 |
|  | No | 20 (52.6) | 244 (68.9) | 3 (75.0) | 1 (100) |
|  | I don’t know | 0 | 2 (0.6) | 0 | 0 |
|  | Not reported | 0 | 4 (1.1) | 0 | 0 |
|  | | | | | |
| Has mental health problems: n (%) | Yes | 13 (34.2) | 95 (26.8) | 4 (100) | 0 |
|  | No | 23 (60.5) | 252 (71.2) | 0 | 1 (100) |
|  | I don’t know | 1 (2.6) | 2 (0.6) | 0 | 0 |
|  | Not reported | 1 (2.6) | 5 (1.4) | 0 | 0 |
|  | | | | | |
| Has been screened for mental health problems in the past year: n (%) | Yes | 12 (31.6) | 44 (12.4) | 3 (75.0) | 0 |
|  | No | 26 (68.4) | 305 (86.2) | 1 (25.0) | 1 (100) |
|  | I don’t know | 0 | 2 (0.6) | 0 | 0 |
|  | Not reported | 0 | 3 (0.8) | 0 | 0 |
|  | | | | | |
| Has nerve problems: n (%) | Yes | 17 (44.7) | 157 (44.4) | 4 (100) | 1 (100) |
|  | No | 20 (52.6) | 192 (54.2) | 0 | 0 |
|  | I don’t know | 1 (2.6) | 3 (0.8) | 0 | 0 |
|  | Not reported | 0 | 2 (0.6) | 0 | 0 |
|  | | | | | |
| Has been screened for nerve problems in the past year: n (%) | Yes | 15 (39.5) | 105 (29.6) | 3 (75.0) | 0 |
|  | No | 23 (60.5) | 247 (69.8) | 1 (25.0) | 1 (100) |
|  | I don’t know | 0 | 1 (0.3) | 0 | 0 |
|  | Not reported | 0 | 1 (0.3) | 0 | 0 |
|  | | | | | |
| Has other diabetes related problems: n (%) | Yes | 8 (21.1) | 17 (4.8) | 0 | 0 |
|  | No | 20 (52.6) | 196 (55.4) | 1 (25.0) | 0 |
|  | I don’t know | 1 (2.6) | 46 (13.0) | 0 | 0 |
|  | Not reported | 9 (23.7) | 95 (26.8) | 3 (75.0) | 1 (100) |
|  | | | | | |
| Has been screened for other diabetes related problems in the past year: n (%) | Yes | 1 (2.6) | 13 (3.7) | 0 | 0 |
|  | No | 22 (57.9) | 228 (64.4) | 2 (50.0) | 0 |
|  | I don’t know | 1 (2.6) | 25 (7.1) | 0 | 0 |
|  | Not reported | 14 (36.8) | 88 (24.8) | 2 (50.0) | 1 (100) |
|  | | | | | |
| Fear of Complications Scale (range: 15−60, higher numbers indicate greater fear) | Mean (SD*) | 22.58 (9.76) | 18.25 (10.39) | 21.00 (11.80) | 15.00^a^ |
|  | | | | | |
| Hypoglycemia Fear Scale (range: 0−108, higher numbers indicate greater fear) | Mean (SD) | 34.00 (17.40) | 20.64 (16.13) | 32.50 (17.41) | 25.00^a^ |
|  | | | | | |
| Diabetes Distress Scale (range: 1−6, higher numbers indicate greater distress) | Mean (SD) | 2.81 (1.23) | 2.23 (1.08) | 2.25 (0.91) | 2.71^a^ |
